# An hiv infection - a problem of quality of life!?

**DOI:** 10.1192/j.eurpsy.2021.1304

**Published:** 2021-08-13

**Authors:** C. Vasilescu, C. Manciuc, C. Dorobat, M. Largu

**Affiliations:** 1 Infectious Diseases, Hospital of Infectious Diseases Iasi, Iasi, Romania; 2 Infectious Diseases, University of Medicine and Pharmacy “Gr. T. Popa” Iasi, Iasi, Romania; 3 Psychology, University of Medicine and Pharmacy “Gr. T. Popa” Iasi, Iasi, Romania

**Keywords:** quality of life, side effects, antiretroviral therapy, HIV/AIDS

## Abstract

**Introduction:**

The quality of life is a multidiimensional and subjective construct, based on the patient’s experience.

**Objectives:**

The objective of this study is to observe if at the HIV - positive patient the quality of life in relation to health is a consequence of disease and treatment and if his perception about the disease changes his ability to have a full and useful life.

**Methods:**

We centralized the data coming from a number of 600 patients registered in the Iasi Regional Center, for a period of 12 months. The side effects reported by the patients emerged from discussions with the infectious diseases specialist and the psychologist.

**Results:**

From 600 pacients, 59% of them were male with mean age of 21.1 years old. Approximately 14% of the patiens had stable jobs, the rest were unemployed or had part-time jobs. 38% came from foster care units of from broken homes. The average number of days of hospitalization was 4 days, 25% of them were at their first scheme, 10% in the seventh-eighth scheme. Among the antiretroviral side effects patients complained nausea and vomiting in 85% of cases, lipodystrophy symptoms in 25% of cases, diarrhea in 15% of the cases; regarding the psychological aspects, 65% of patients showed an above level of anxiety, 40% had depressive manifestations, 10% had specific obsessions-compulsions and 10% neurotic and hysterical tendencies.

**Conclusions:**

We need a close collaboration between the infectious diseases specialist and the psychologists in order to enhance the quality of life of the HIV patient.

